# Editorial: Deciphering Phagocyte Functions Across Different Species

**DOI:** 10.3389/fcell.2021.712929

**Published:** 2021-07-30

**Authors:** Yi Feng, Marc S. Dionne, Efstathios G. Stamatiades, Katrin Kierdorf

**Affiliations:** ^1^UoE Centre for Inflammation Research, Queen's Medical Research Institute, University of Edinburgh, Edinburgh, United Kingdom; ^2^Medical Research Council Centre for Molecular Bacteriology and Infection and Department of Life Sciences, Imperial College London, London, United Kingdom; ^3^Charité University Medical Centre, Institute of Microbiology, Infectious Diseases and Immunology, Berlin, Germany; ^4^Faculty of Medicine, Institute of Neuropathology, University of Freiburg, Freiburg, Germany; ^5^Center for Integrative Biological Signaling Studies Centre for Integrative Biological Signalling Studies, University of Freiburg, Freiburg, Germany; ^6^Faculty of Medicine, Center for Basics in NeuroModulation (NeuroModulBasics), University of Freiburg, Freiburg, Germany

**Keywords:** phagocyte, macrophage, model organism, innate immunity, species

Specialized innate immune cells are found across all metazoan organisms. Foundational experiments by Elie Metchnikoff revealed the existence of innate phagocytes which efficiently clear invading pathogens or cell debris in the sea star larva (Chang, 2009). The concept of phagocytic immune cells is conserved across insects to mammals and their counterparts are found in *Drosophila*, zebrafish, or mammals. These cells seem to serve two major functions: the clearance of invading pathogens and cell debris, as well as the release of immunomodulatory molecules and growth factors (Kierdorf and Dionne, 2016). Their functions cover a variety of tasks from combating infections or cancer to maintaining tissue homeostasis and repair.

This Research Topic contains a collection of original research articles and literature reviews pointing toward the importance of understanding phagocyte function in a cross-species approach, but also highlighting the necessity and elegance of experimental model organisms in biomedical research. More and more studies reveal that immune functions and ontogeny of many phagocyte subtypes are conserved across species (Gold and Brückner, 2014). By studying phagocyte function in different species we can obtain new insights into the innate immune function of human phagocytes such as granulocyte, monocytes, dendritic cells and macrophages (Geirsdottir et al., 2019; Zindel et al., 2021). Within this Research Topic Miah et al. outline the collective findings on prenatal human mononuclear phagocyte development in different organs and their role in embryonic and fetal organ development and to compare them to the more detailed data already obtained on phagocyte development in transgenic mouse models. They also highlight new data collected from single-cell multi-omic approaches and next-generation *ex-vivo* organ-on-chip models which could guide future studies into understanding human phagocyte development.

Depending on host tissue, phagocytes represent a heterogenous range of subtypes and functional diversity. In their study, Park et al. identify a new adherent intestinal phagocyte population in Atlantic salmon which has high phagocytic activity and expresses several macrophage specific genes. In a detailed review, Portilla et al. summarize the current knowledge on intestinal macrophages from different species. Starting with a description of the development and differentiation of phagocytes in different species during evolution, they further describe tissue specification of vertebrate macrophages and finally the highly adapted specialization of different intestinal macrophage subsets. Highlighting the complexity of the interplay of resident tissue macrophages with their niche, they also point toward the necessity to not only decipher heterogeneity across different phagocytes but also understand their interaction network within their specific tissue niche.

Many immune defense pathways of phagocytes are conserved from invertebrates to vertebrates. Moghadam et al. describe recent data on the role and function of reactive oxygen species (ROS) produced by phagocytes from flies to humans. Here they also point to the importance of the collected data from model organisms which help to clarify the function of ROS and ROS deficiency during human disease. Bush et al. describe and compare the transcriptomic profile of sheep bone marrow derived macrophages (BMDMs) with BMDMs from other larger animals and rodents upon lipopolysaccharides (LPS) stimulation. This analysis highlights conserved transcriptional profiles between the species in genes clusters such as cell surface receptors and the endosome-lysosome pathway, but also differences in the induction of arginine metabolism and nitric oxide production.

A wide range of models for chronic disease, cancer and infection have been established in various model organisms and help us to understand phagocyte function *in vivo* (Dionne and Schneider, 2008; Feng and Martin, 2015). Kumar et al. present a new tool to study phagocytes in insect immunity by depleting phagocytes in *Drosophila melanogaster* and *Aedes aegypti* with clodronate liposomes. This depletion study demonstrates the central function of phagocytic immune cells in defense against invading pathogens. *Drosophila melanogaster* has emerged as a versatile tool to study immunometabolism and the interaction between immune signaling and adaptations in metabolism for example during infection but also how metabolic changes can trigger an immune response. Bajgar et al. summarize collective knowledge on the immunometabolism of hemocytes in *Drosophila* and discuss new hypotheses based on the available data. Using a mouse model for choroidal neovascularization (CNV) during age-related macular degeneration, Schlecht et al. demonstrate their findings on the role of macrophage-derived *secreted phosphoprotein-1* (*Spp1*) during CNV. Combining subsequent gene expression analysis of resident retinal microglia and *in vivo* inhibition of Spp1, they provide evidence that the Spp1 pathway could be a promising new target to modulate CNV in human patients.

Advances in *in vivo* imaging techniques now allow tracing of phagocytes in the living organism (Nimmerjahn et al., 2005; Stamatiades et al., 2016). The zebrafish is an excellent model system due to the high transparency of larvae and the availability of many transgenic reporter lines. Hu et al. use live imaging in zebrafish larvae to follow the migration behavior of neutrophils and macrophages in response to tail wounding. Interestingly, they find *toll like receptor 2* (*tlr2*) and *myeloid differentiation primary response 88* (*myd88*) are involved in modulating directional migration of distant neutrophils and macrophages to the wound. Detailed analysis of neutrophils and macrophage migration behavior revealed that the Tlr2-MyD88 pathway controls directional persistence and the migration speed of recruited cells. Future studies are now needed to further elucidate the mechanism as to how Tlr2-MyD88 signaling pathway cross talks to chemokine signaling to control neutrophil and macrophage responses to the wound signal. Recently new high-dimensional, high-throughput techniques, such as single cell RNA-sequencing (scRNA-seq) have allowed us to gain further insight on the heterogeneity of phagocytes in different species including embryonic and larval hemocytes in *Drosophila melanogaster* (Cattenoz et al., 2020; Cho et al., 2020; Tattikota et al., 2020). In this context, Cattenoz et al. compare the collective data from these three studies and identify eight hemocyte subgroups within all three datasets which are associated to distinct functions during embryonic and larval stages. Their comparison further highlights the distinct expression profile of larval hemocytes in peripheral tissues. The heterogenous functions of *Drosophila* macrophages in different organs during development, but also homeostasis, are summarized by Mase et al. In their contribution they give an overview of the recent knowledge on organ specific macrophage functions in *Drosophila*.

In the immunological field, there are few cross-species comparisons of phagocyte function. To our knowledge, the exchange of data between groups studying phagocyte function in different model organisms is most limited. How tissue-derived signals regulate phagocyte function is also barely understood. The articles and reviews collected for this Research Topic show that comparison and analysis of phagocyte function in different organisms might contribute to our knowledge about the innate immune system and help to understand immune responses during different diseases such as cancer, infection or metabolic disease.

## Author Contributions

All authors listed have made a substantial, direct and intellectual contribution to the work, and approved it for publication.

## Conflict of Interest

The authors declare that the research was conducted in the absence of any commercial or financial relationships that could be construed as a potential conflict of interest.

## Publisher's Note

All claims expressed in this article are solely those of the authors and do not necessarily represent those of their affiliated organizations, or those of the publisher, the editors and the reviewers. Any product that may be evaluated in this article, or claim that may be made by its manufacturer, is not guaranteed or endorsed by the publisher.
